# Zero-Shot Sketch-Based Image Retrieval Using StyleGen and Stacked Siamese Neural Networks

**DOI:** 10.3390/jimaging10040079

**Published:** 2024-03-27

**Authors:** Venkata Rama Muni Kumar Gopu, Madhavi Dunna

**Affiliations:** Department of Electrical, Electronics and Communication Engineering (EECE), Gitam School of Technology, Gitam Deemed to be University, Rushikonda, Visakhapatnam 530045, India; mdunna@gitam.edu

**Keywords:** sketch-based image retrieval, SSiNN, stacked Siamese neural network, domain gap, ZS-SBIR

## Abstract

Sketch-based image retrieval (SBIR) refers to a sub-class of content-based image retrieval problems where the input queries are ambiguous sketches and the retrieval repository is a database of natural images. In the zero-shot setup of SBIR, the query sketches are drawn from classes that do not match any of those that were used in model building. The SBIR task is extremely challenging as it is a cross-domain retrieval problem, unlike content-based image retrieval problems because sketches and images have a huge domain gap. In this work, we propose an elegant retrieval methodology, StyleGen, for generating fake candidate images that match the domain of the repository images, thus reducing the domain gap for retrieval tasks. The retrieval methodology makes use of a two-stage neural network architecture known as the stacked Siamese network, which is known to provide outstanding retrieval performance without losing the generalizability of the approach. Experimental studies on the image sketch datasets TU-Berlin Extended and Sketchy Extended, evaluated using the mean average precision (mAP) metric, demonstrate a marked performance improvement compared to the current state-of-the-art approaches in the domain.

## 1. Introduction

Sketch-based image retrieval (SBIR) [1,2,3,4] constitutes a specific subset within the wider spectrum of content-based image retrieval (CBIR) problems. Content-based image retrieval (CBIR) refers to the process of retrieving relevant images from a large database based on the contents and intent of the input query images rather than manually entered metadata or keywords. In SBIR, the system is presented with ambiguous sketches as input queries and is tasked with retrieving corresponding matches from a database composed of natural images.

The evolution of SBIR has closely paralleled advancements in image processing [5,6], computer vision, and machine learning. Originating in the late 1990s as an offshoot of CBIR, early SBIR systems focused on basic shape matching using simple feature extraction techniques [7]. The early 2000s saw the integration of more sophisticated feature descriptors like SIFT and HOG [2], enhancing the accuracy of sketch-to-image matching. The advent of deep learning, particularly with convolutional neural networks (CNNs), in the 2010s marked a significant milestone, drastically improving the ability to bridge the domain gap between sketches and natural images. Vehicle re-identification [8] and person re-identification [9] are closely related to image recognition and play a crucial role in security, surveillance, and traffic management applications. Recent developments have delved into zero-shot learning and cross-domain retrieval, pushing the boundaries of SBIR in handling diverse and unseen sketch categories. Today, SBIR [10,11] continues to evolve, integrating with emerging technologies and expanding its application scope.

Image retrieval using sketches as query input is gaining more practical significance, particularly with the rise of touch-based devices and the increasing demand for accessibility features. A practical application of SBIR involves utilizing free-hand sketches as search queries, particularly when textual search methods are impractical or when language barriers are present. Highlighting the versatility of SBIR techniques, the following are some key applications:

- Law enforcement and forensic art [12]: In law enforcement, SBIR can help match sketches of suspects or missing persons with photographic databases.
- E-commerce and online retail [13]: SBIR can be used in online shopping platforms to allow users to sketch an item they wish to purchase. This is particularly useful when shoppers are unsure of the technical name of the item but can draw it.
- Digital art and graphic design [14]: Artists and designers can use SBIR to find reference images based on a rough sketch. This is useful in creative processes where visualizing an idea is easier through drawing than describing it in words.
- Education and research [15]: In educational settings, SBIR can assist students and researchers in finding scientific diagrams or historical images based on hand-drawn sketches. This can be particularly useful in fields like archaeology, history, or biology.
- Medical imaging [11]: SBIR can be used in medical diagnostics by allowing doctors to sketch symptoms or conditions and retrieve similar medical images or case studies. This could be particularly useful in dermatology or radiology.
- Cultural heritage and museums [16]: Museums and cultural institutions can use SBIR to help visitors connect with artworks or artifacts. Visitors could sketch an artifact or art piece they are interested in and receive information about similar items in the museum’s collection.
- Architecture and interior design [17]: Architects and interior designers can use SBIR to find building designs, interior decor ideas, or furniture based on sketches. This can streamline the process of translating conceptual sketches into concrete plans or finding matching furniture and decorations.

SBIR presents a significant challenge because of the substantial domain gap between the sketch input and image database, thereby transforming it into a cross-domain image retrieval issue. Sketches are notably abstract and devoid of key features typically leveraged in traditional content-based image retrieval, such as shape features, color attributes, texture, and structural properties. This absence of features further compounds the complexity of SBIR, rendering it a more intricate task compared to standard CBIR [18,19] activities. Delving a bit deeper into specific challenges and limitations faced by SBIR, Different people have different sketching styles, and even the same person may sketch differently at different times. This variability can lead to inconsistencies in how objects are represented, making it difficult for SBIR systems to accurately match sketches with images. There is a significant domain gap between the high-dimensional data of photographs and the low-dimensional, abstract nature of sketches. Sketches can be symbolic or abstract [20], not always representing real-world objects accurately or realistically. This abstraction poses a challenge in matching these sketches with real images, especially when the sketches represent conceptual ideas rather than concrete objects. Sketches may not always maintain consistent scale or orientation relative to the actual objects they represent. An SBIR system needs to be robust to such variations, which adds complexity to the retrieval process. For SBIR systems to be practical, especially in commercial applications, they need to offer real-time or near-real-time performance. Processing sketches and searching through large image databases efficiently are computationally demanding tasks that require optimized algorithms and hardware. Understanding the semantic meaning of a sketch [21] (what object or concept it represents) is a complex task. This is particularly challenging when sketches are vague or when the same sketch could represent multiple objects. An SBIR system trained on one dataset may not perform well on another due to differences in image types, sketch styles, and object categories. Ensuring that these systems generalize well across different datasets is a significant challenge. In realistic scenarios, as databases expand to include new image categories, an SBIR system may lack prior information about these novel classes. The task of retrieving such images, which were not represented in the original system design, is referred to as zero-shot sketch-based image retrieval (ZS-SBIR) [22,23,24]. This task introduces an additional layer of complexity due to the inherent knowledge gap associated with zero-shot samples, posing an even greater challenge to the system. The fundamental challenge in zero-shot learning [25] is the absence of training examples for unseen classes. The model must infer knowledge about these classes from the data it has, which can be difficult if the unseen classes are significantly different from the seen ones. The model must generalize from the seen classes to the unseen ones. This requires an understanding of underlying patterns and features that are common across classes, which is a complex task, especially when the unseen classes diverge significantly from the seen ones. Bridging the semantic gap between low-level features extracted from data and high-level class concepts is challenging. The model must understand and utilize abstract, semantic relationships without having direct examples of those relationships. Models trained on a specific set of classes may develop biases toward those classes, leading to poor performance when encountering new classes. This domain shift is a significant hurdle in ensuring that the model performs well on both seen and unseen classes. Models need to be specific enough to accurately categorize seen classes but also general enough to adapt to unseen classes. Finding this balance is challenging, as overfitting to the seen classes can reduce the model’s ability to generalize. As the number of classes increases, the computational complexity can grow significantly. Ensuring that the model scales efficiently and maintains performance with a growing number of classes is a challenge.

Our contributions:

1. Propose a novel approach for the ZS-SBIR problem through the segregation of domain gap reduction and image-retrieval stages.
2. Propose the mathematical formulation of the StyleGen approach, illustrating various loss functions involved in training an effective model for domain gap reduction between sketches and images.
3. Presents the neural network architectures for the StyleGen model, which comprises generator and discriminator blocks.
4. Provides an adaption of the latest SSiNN [26] architecture for image retrieval to maximize the overall system’s retrieval performance.
5. Presents the datasets used in the experimental study and the performance metrics used for the evaluation and comparison with the existing approaches.
6. Presents a comprehensive presentation of experimental results, illustrating the effectiveness of our approach in ZS-SBIR scenarios.

## 2. Related Work

In this section, we will provide a brief overview of the research literature related to SBIR (sketch-based image retrieval), ZSL (zero-shot learning), and ZS-SBIR.

### 2.1. Sketch-Based Image Retrieval

SBIR approaches primarily focus on addressing the challenges associated with the domain gap between sketches and images. They aim to develop techniques that can effectively capture and represent the visual features of sketches and images, devise matching algorithms to measure their similarity, and design retrieval strategies to accurately retrieve relevant images based on user-provided sketches.

Ming Zh et al. [27] proposed a gradually focused bilinear attention model to improve fine-grained image retrieval based on sketches by accurately highlighting representative local positions and using weighted bilinear coding for more discriminative feature representations. Deep network architectures for sketch-based image retrieval (SBIR) employing convolutional neural networks (CNNs) with multi-stage regression are proposed and evaluated in the research by Bui et al. [28]. The authors investigate their networks’ capacity to generalize across multiple object categories using minimal training data, as well as methodologies for weight sharing, preprocessing, data augmentation, and dimensionality reduction. They describe a hybrid multi-stage training network for improving performance by combining contrastive and triplet networks. The work by Zhou et al. [29] proposed a deep learning approach that dealt with sketch data scarcity by incorporating a method for sketch augmentation that generates additional sketches from existing data by removing, adjusting, and rotating strokes. The proposed approach utilizes a multi-domain learning technique that employs a couple of Siamese CNNs that pair 2D shape images and sketches in conjunction with a joint Bayesian metric to maximize inter-class similarity and minimize intra-class similarity. The study by Niteesh et al. [30] proposed image preprocessing and deep learning-based methods to fix sketch-based image retrieval (SBIR) systems that do not have enough semantic knowledge. The Canny edge detection method makes a binary image of the edges of the natural image. CNN models that are built on ImageNet pull out deep features. Rocchio’s method is used to provide relevant feedback for gap identification.

### 2.2. Zero-Shot Learning

Zero-shot learning is a method that aims to recognize unseen categories by using a shared visual-semantic function. The paper by Xian et al. [31] addresses the need for a unified benchmark in zero-shot learning and proposes a new benchmark by defining evaluation protocols and data splits. It emphasizes the importance of comparable and reliable results in the field. The study by Li et al. [32] presents a zero-shot learning strategy that simultaneously learns visual prototypes and maintains semantic consistency across visual and semantic domains, yielding much better outcomes.

### 2.3. Zero-Shot Sketch-Based Image Retrieval

ZS-SBIR refers to the task of retrieving relevant images from a database using a sketch as a query when there are no examples of that specific class in the training set. The GTZSR framework [33] employs a graph transformer to maintain the semantic space class topology while transmitting the visual space’s class context graph. It attempts to narrow the domain gap between image and sketch features by minimizing the Wasserstein distance between them. The ACNet framework [23] employed a two-module approach in which a retrieval module directs the synthesis module to produce a variety of images that eventually converge to the domain of the photos. The paper by Dutta et al. [24] introduced “StyleGuide”, a unique retrieval method for ZS-SBIR that employs style-guided fake-image generation.

## 3. Proposed StyleGen for ZS-SBIR

**Figure 1:**
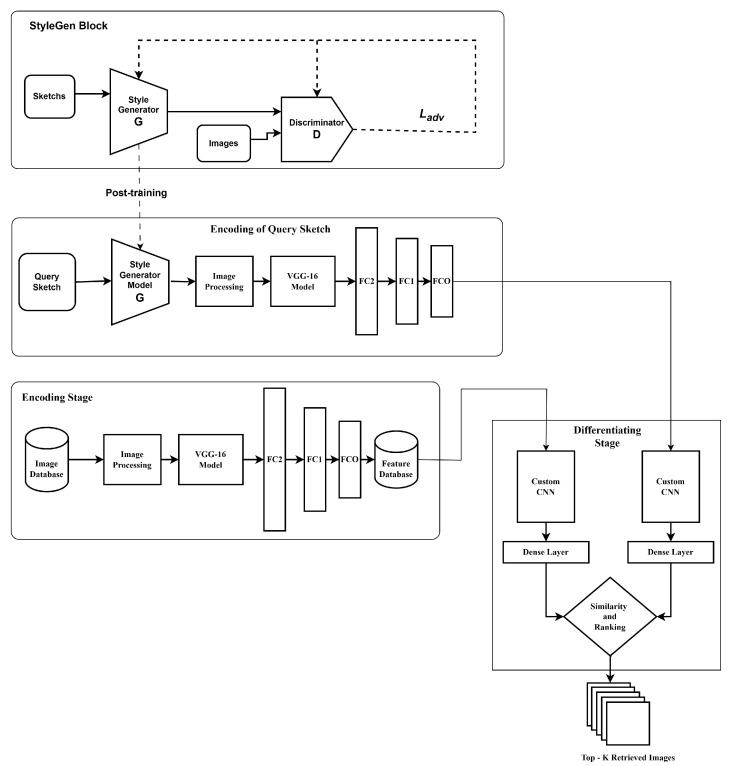
The StyleGen block contains a GAN network, which is trained to generate images equivalent to the input sketches in the domain of the images. The trained generator model is fed with the query sketch to generate its corresponding StyleGen image. This image is encoded using the encoder block of the SSiNN and is differentiated against the encoded versions of the database images. The differentiating stage ranks and furnishes the top K relevant images.

In this paper, we put forward an innovative technique for ZS-SBIR using a combination of StyleGen and a stacked Siamese Neural network (SSiNN). Our method leverages the power of generative adversarial networks (GANs) to synthesize photographic images from sketches and then employs a stacked Siamese network to perform efficient image retrieval. A thorough evaluation of our methodology is conducted using benchmark datasets, wherein it is shown to outperform current techniques. Our findings highlight the potential of our method to revolutionize the field of sketch-based image retrieval and open up new avenues for research in this area. In Figure 1, we present a high-level block diagram that provides a schematic representation of the proposed StyleGen approach, elucidating its core components and their interrelationships.

### 3.1. Problem Formulation

The problem formulation for dividing the dataset into training and test datasets for ZS-SBIR can be expressed as follows: Given a dataset consisting of sketches and corresponding images, the goal is to partition the dataset into two subsets, $D_{train}$ and $D_{test}$, such that $D_{train}$ contains a set of sketches and corresponding images from a subset of the classes in the dataset. These sketches and images are used to train the ZS-SBIR system. $D_{test}$ contains the remaining set of sketches and corresponding images from the remaining classes in D. These sketches are used as queries to evaluate the performance of the ZS-SBIR methodology. The partitioning of the dataset should be done in such a way that the classes in $D_{train}$ and $D_{test}$ are disjoint, i.e., no class should appear in both sets. Moreover, the partitioning should ensure that there is a sufficient number of examples from each class in both $D_{train}$ and $D_{test}$ to ensure a fair evaluation of the system’s performance. The quality of the partitioning can have a significant impact on the accuracy of the system’s performance and, therefore, should be carefully considered during system development.

The methodology can be divided into two phases, each addressing a sub-problem of the overall ZS-SBIR. Phase I focuses on domain gap reduction between the sketches and images. The effectiveness of this phase determines the overall efficacy of the system, as it minimizes the discrepancy between the feature space representations of sketches and images. Phase II focuses on the actual feature space representation, which effectively encodes the images and sketches so as to maximize the retrieval performance.

Let Y denote the set encompassing all labels present within the dataset. Partition Y in to 2 disjoint sets $Y_{train}$ and $Y_{test}$. Hence

(1) $$\begin{array}{c} Y_{train}\cap Y_{test}=\phi \end{array}$$

(2) $$\begin{array}{c} Y_{train}\cup Y_{test}=Y \end{array}$$

Let $D_{train}$ and $D_{test}$ are the Train and Test datasets, respectively. Both training and test sets are a combination of sets of images and sketches.

(3) $$\begin{array}{c} D_{train}=X_{train}\cup S_{train} \end{array}$$

(4) $$\begin{array}{c} D_{test}=X_{test}\cup S_{test} \end{array}$$

### 3.2. No Pair Assumption

**Figure 2:**
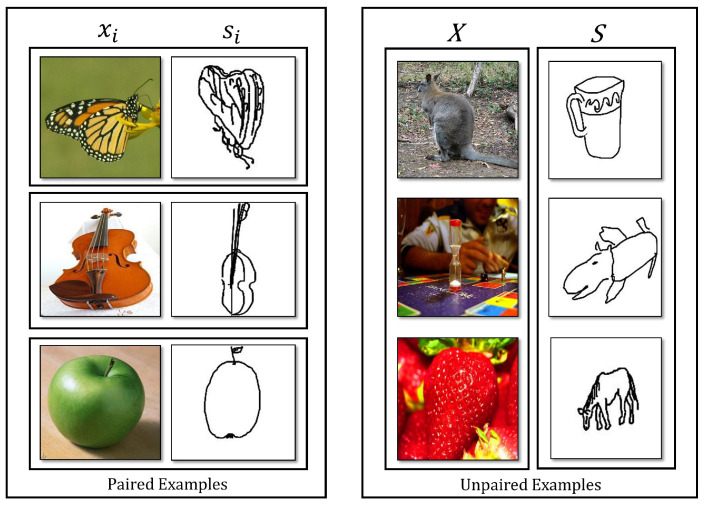
The image is divided into two distinct sections. On the left side, there are “paired examples”, which consist of images and sketches that are presented in matching or corresponding pairs, demonstrating a relationship or connection between them. Each pair could be similar in appearance, function, or concept, indicating a clear link or duality. On the right side, the “unpaired examples” are displayed. These consist of images and sketches that stand alone without an apparent match or counterpart. They may be varied in appearance, function, or concept, lacking the obvious pairing found in the examples on the left.

In the realm of ZS-SBIR, the “no pair assumption” signifies that during the training phase of the retrieval system, there are not any directly corresponding or “paired” sketches and images available. The no-pair assumption is fundamental in many SBIR methods, particularly in zero-shot SBIR, where the goal is to retrieve images of classes not seen during training. This assumption allows the SBIR system to learn a mapping from the sketch space to the image space that can be generalized to unseen classes. If paired sketches and images were used during training, the SBIR system could simply learn to memorize the paired examples rather than learning a generalizable mapping between the sketch and image spaces. This would result in poor performance in novel classes during testing. An example of paired and unpaired cases is illustrated in Figure 2.

### 3.3. Neural StyleGen for Domain Gap Reduction

StyleGen is the process of transferring the domain characteristics of images onto the sketches while preserving their intents. We will use generative neural network models to learn the domain style and content representations of the images and then infuse them into the sketch intent to produce a generated image with the desired style and domain. We use CycleGAN architecture [34] for the StyleGen part of the ZS-SBIR system proposed. CycleGAN architecture learns the features of one set of images and applies them to another set of images without the need for paired training examples. CycleGAN uses two sets of GANs, one for each direction of translation, each consisting of a generator network that maps images from one domain to another and a discriminator network that attempts to distinguish between the generated images and the real images in the target domain. Collaboratively, the generator and discriminator networks undergo adversarial training, during which the generator attempts to generate near-real images with the intention of deceiving the discriminator. On the other hand, the discriminator endeavors to accurately differentiate between real and generated images.

### 3.4. StyleGen Approach

Let $X_{train}$ and $S_{train}$ be the image set and sketch set, respectively, from the training data.

**Definition** **1.** *The StyleGen function can be defined as SG, which transforms the sketch images into image-like StyleGen images.*

(5) $$\left\{\forall s_{i}\in S_{\mathrm{train}},\exists \;\mathrm{SG}\;\mathrm{such}\;\mathrm{that}\;{\mathrm{sg}}_{i}=\mathrm{SG}\left(s_{i}\right)\right\}$$

*where $\mathrm{SG}:S_{\mathrm{train}}\to X_{\mathrm{train}}$*

For simplicity, let us denote *X* as the image set and *S* as the sketch set. Let *G* be the generator function, which transforms sketches into images. The purpose of *G* is to take the samples from the sketch database S and to generate corresponding StyleGen images.

(6) G:S→X

Let $G^{\prime }$ be the generator function, which does the transformation in the reverse direction.

(7) $$G^{\prime }:X\to S$$

The aim of the learning functions above can be defined as follows:

(8) $$\begin{array}{c} G’(G(s))\approx s \end{array}$$

(9) $$\begin{array}{c} G(G’(x))\approx x \end{array}$$

Let *D* be the discriminator function; we define two discriminator functions, $D_{s}$ and $D_{x}$. The purpose of these functions is to distinguish real and generated images in their respective domains. Discriminators in CycleGAN provide feedback to the generators and help them fine-tune themselves, thus enhancing the generated images’ quality. The discriminator acts as a sort of "adversary" that challenges the generators to produce better and more realistic images. The discriminator, $D_{s}$, differentiates between *s* and the transformed images, G’(x), whereas $D_{x}$ differentiates *x* and G(s).

### 3.5. Adversarial Loss

The adversarial loss is the objective function used to train the GAN. It is based on the idea that the generator network should be able to produce synthetic data that is indistinguishable from real data. The adversarial loss is evaluated as the cross-entropy of the discriminator’s output relative to the true label, where the true label is 1 for real data and 0 for synthetic data. In other words, the generator network attempts to minimize the adversarial loss by generating synthetic data that maximally fool the discriminator into believing that it is real. The discriminator network, on the other hand, attempts to maximize the adversarial loss by correctly classifying real and synthetic data. Overall, the adversarial loss plays a crucial role in the GAN architecture, as it drives the competition between the generator and discriminator networks and encourages the generator to produce high-quality synthetic data. Let the data distribution of sketch data be q(s), and that of image data be p(x).

(10) $$\begin{array}{c} s∼q(s) \end{array}$$

(11) $$\begin{array}{c} x∼p(x) \end{array}$$

(12) $$\begin{array}{cc} L_{\mathrm{adv}}\left(G,D_{X}\right) & =E_{p}\left[\ln D_{X}\left(x\right)\right]+E_{q}\left[\ln\left(1-D_{X}\left(G\left(s\right)\right)\right)\right] \end{array}$$

(13) $$\begin{array}{cc} L_{\mathrm{adv}}\left(G^{\prime },D_{S}\right) & =E_{q}\left[\ln D_{S}\left(s\right)\right]+E_{p}\left[\ln\left(1-D_{S}\left(G^{\prime }\left(x\right)\right)\right)\right] \end{array}$$

The overall adversarial loss function is as follows:

(14) $$L_{\mathrm{adv}}=L_{\mathrm{adv}}\left(G,D_{X}\right)+L_{\mathrm{adv}}\left(G^{\prime },D_{S}\right)$$

### 3.6. Cycle Consistency Loss

The second component is the cycle consistency loss, which ensures that the generator produces images that can be transformed back to the original domain without losing information. It is defined as follows:

(15) $$L_{\mathrm{cyc}}\left(G,G^{\prime }\right)=E_{q}\left[\left|\left|G^{\prime }\left(G\left(s\right)\right)-s\right|\right|\right]+E_{p}\left[∥G(G^{\prime }\left(x\right))-x∥\right]$$

### 3.7. Identity Loss

The identity loss ensures that the generator network is able to produce outputs that are not only realistic but also retain some of the original characteristics of the input data. By incorporating identity loss into the GAN training process, the generator is encouraged to produce outputs that are both realistic and retain the original properties of the input data. This can improve the quality and fidelity of the generated data and can also help mitigate the mode collapse problem, where the generator produces only a limited set of outputs.

(16) $$L_{\mathrm{identity}}=E_{x}\left[{\left|G(x)-x\right|}_{1}\right]+E_{s}\left[{\left|G^{\prime }\left(s\right)-s\right|}_{1}\right]$$

where:

- G(.) denotes the sketch-to-image generator function.
- *x* indicates an image sample from the image dataset.
- $E_{x}$ denotes the expected value, evaluated over image data distribution.
- $G^{\prime }\left(.\right)$ denotes the image-to-sketch generator function.
- *s* indicates a sketch sample from the sketch dataset.
- $E_{s}$ denotes the expected value, evaluated over sketch data distribution.

### 3.8. Overall Objective Function

The overall combined objective function is as follows:

(17) $$L_{\mathrm{objective}}=L_{\mathrm{adv}}+\alpha L_{\mathrm{cyc}}\left(G,G^{\prime }\right)+\beta L_{\mathrm{identity}}$$

Here, α and β are hyperparameters, where α denotes cycle-consistency loss weight and β denotes identity loss weight.

For our experiments: α=10 and β=0.5.

### 3.9. Network Architecture of Generator

The generator is implemented using a deep convolution network for transforming input images into corresponding target representations. Our network initiation incorporates a 2D convolution layer featuring a 7×7 kernel, Instance normalization, and ReLU activation follow. As we delve deeper into the network, layers are structured to systematically augment the channel depth and concurrently down-sample the spatial dimensions. This is achieved through 3×3 convolutional filters, together with a stride of 2. These downsampling blocks are complemented with instance normalization and ReLU activation functions. We implement two such downsampling blocks in the generator design. Central to our design is the incorporation of eight residual blocks. Every block is structured with a pair of convolutional layers, both furnished with 3×3 kernels and further enhanced by instance normalization and ReLU activations. A defining characteristic of these blocks is the summation of the incoming input with the processed output, enabling the network to discern and adapt to residual functions. In the advanced segments of our generator, we employed transposed convolutions to methodically revert the downsampling operations. This ensures a progressive recovery of spatial resolutions while concurrently tapering the channel depth. Culminating our design, the final output generation is entrusted to a 7×7 convolutional layer, which is succeeded by a tanh activation, thus ensuring that all output values adhere to the range [−1,1]. The network architecture and the parameters are presented in Table 1.

### 3.10. Network Architecture of Discriminator Neural Network

The discriminator network adapted from [35] comprises a series of convolutional layers, progressively increasing in channels from 3 to 512. A combination of LeakyReLU activation and instance normalization was adopted across layers for stability and performance. The topology of the network is presented in Table 2.

### 3.11. Training the Networks of StyleGen Phase

The StyleGen framework employs the generator and discriminator networks described in previous sections. For the image retrieval application, we are exclusively concerned with the forward process of transforming sketches into images. The inverse process—from images back to sketches—is not required because the major goal of this process is to combat the domain gap problem, thereby restyling sketches into the image domain, which can then be used for image retrieval tasks. The dataset is partitioned using the approach specified in the current section’s problem formulation sub-section. The partition specifics for the datasets utilized will be expanded on in the experimental results section. Once the training is complete, the generator model can be used for transforming the sketches to the image domain. The generator model, which we call the StyleGen model, is used to generate images equivalent to the query sketches. By doing this, the SBIR task would now be boiled down to a CBIR task where the input query is an image.

### 3.12. Stacked Siamese Neural Network for Image Retrieval

We use a CBIR technique from [26] for the retrieval task. SSiNN is a two-stage CBIR system that uses a pre-trained model customized to the dataset of study for encoding the input images and uses a Siamese neural network on the encoded images to differentiate and rank the database images for the effective retrieval of the intended images. To train the SSiNN, we use the image sub-set from the training partition of the partitioned dataset. To train the first stage of the SSiNN, we employ VGG-16 architecture and use the image sub-set of the training data. To be precise, the overall dataset is partitioned into training and test datasets, with disjoint classes as described in the partition strategy. From the training dataset, we only use the image sub-set and exclude the sketch sub-set for the training process. To train the second stage, the model of the first stage is used to encode the training dataset, and the encoded vectors are used to train the second stage.

1. Input the StyleGen model with the query sketch to obtain the image domain equivalent representation.
2. To extract the latent space representations of the database Images, run them through the first stage of the SSiNN.
3. Run the StyleGen output from step 1 through the first stage of the SSiNN to acquire the sketch’s latent space representation.
4. Now, pass the sketch representation through one input of the Siamese neural network and the latent space representations from the database through the other input of the Siamese neural network.
5. Rank the outputs and provide the top-K images corresponding to the representations as the SBIR system’s output.

#### Simplified Decision-Making Process

In this subsection, we present a simplified overview of the decision-making process. The intricacies of network training and the detailed architecture have been extensively covered in previous sections with a higher degree of technical specificity. Additionally, to maintain a streamlined representation, this subsection does not delve into the optimizations employed, such as the storage of latent space representations in the database.

- The SSiNN model, employed for the retrieval operation, is a two-input model. Henceforth, this model shall be designated as the retrieval model.
- The first input channel of the retrieval model is ingested with images from the database, from which relevant images are to be extracted.
- The second input channel of the retrieval model is allocated for processing the image representation derived from the sketch-based query.
- The transformation of the sketch into its image representation is facilitated by the StyleGen model.
- Subsequently, the retrieval model computes similarity metrics across the dataset images, ranking them based on these scores. Images attaining the highest similarity metrics are identified as the most relevant matches to the input sketch query.

## 4. Experimental Results

This section provides an exposition of the experimental outcomes obtained from the employed methodology. We evaluate the effectiveness of our approach using two benchmark datasets: “Sketchy Extended” and “TU-Berlin Extended”. We present the retrieval performance and compare it to existing approaches.

### 4.1. Datasets

The “Sketchy Extended” dataset comprises a total of 75,481 sketches and 73,002 photos, including 60,502 images from the ImageNet dataset and 12,500 sourced from [14]. With 125 categories, the dataset encompasses a diverse array of everyday objects, animals, vehicles, and more. To conduct experiments on ZS-SBIR, the dataset is partitioned [22], such that the data sourced from ImageNet, distributed over 21 classes, are allocated for testing, while the remaining data from other classes are utilized for training purposes.

The “TU-Berlin Extended” dataset, as described in [36], consists of 20,000 sketches across 250 object classes. It also includes 204,070 photo images furnished by Liu et al. [3]. The dataset is partitioned into training and test sets under the zero-shot setting by utilizing the partitioning protocol illustrated in [37]. For testing, 30 classes are randomly selected, ensuring each class has a minimum of 400 photo images, and the rest of the classes are used for training purposes.

The choice to utilize the TU-Berlin and Sketchy Extended datasets was made after thoughtful consideration, keeping in mind their rich diversity, the wide array of categories, and the varied drawing styles they encompass. These characteristics are pivotal for assessing the generalization ability of ZS-SBIR systems in a robust manner. Moreover, the dataset, with its comprehensive set of photo-sketch pairs, presents an unparalleled opportunity to delve into cross-modal retrieval challenges within a controlled yet demanding environment, which is crucial for pushing the boundaries of zero-shot learning research. The decision was driven by the objective of thoroughly evaluating our model’s adaptability and effectiveness across diverse and rigorous conditions, thereby ensuring its reliability and practical utility in scenarios reflective of the real world. Leveraging these datasets allows for meaningful benchmarking against the current state-of-the-art, contributing significantly to the advancement of knowledge in the field of ZS-SBIR.

### 4.2. Evaluation Metric

To assess the performance of ZS-SBIR systems in this research, we utilize the mean average precision (mAP) as our evaluation metric. The mAP offers a single scalar value representing the overall average precision across different queries. It is calculated by first determining the average precision (AP) for each sketch query and then computing the mean of these AP values. The mAP effectively encapsulates the system’s overall retrieval performance, making it a reliable and widely accepted metric for such evaluations.

**Average Precision (AP)**: The average precision for a query is the mean of the precision scores obtained for each relevant item in the retrieved list, indicating the precision of the system at the rank of that item.

(18) $$\mathrm{AP}=\frac{\sum_{k=1}^{N}P\left(k\right)\times r\left(k\right)}{\mathrm{Number}\;\mathrm{of}\;\mathrm{Relevant}\;\mathrm{Items}}$$

where

- P(k) signifies precision at rank *k*.
- *N* is the cardinality of the retrieved list.
-
$r\left(k\right)=\left\{\begin{array}{cc} 1 & \mathrm{if}\;\mathrm{the}\;\mathrm{item}\;\mathrm{is}\;\mathrm{relevant},\\ 0 & \mathrm{otherwise}. \end{array}\right.$

**mAP@all (Mean average precision at all ranks)**: This metric computes the mean of the average precision (AP) scores across all queries, with each AP score calculated using the entire ranked list of retrieved items. Its formula is given by the following:

(19) $$\mathrm{mAP}@\mathrm{all}=\frac{\sum_{q=1}^{\left|Q\right|}{\mathrm{AP}}_{q}}{\left|Q\right|}$$

where:

- |Q| is the cardinality of the query list.
- ${\mathrm{AP}}_{q}$ is the average precision for the *q*th query calculated over the entire retrieved list.

**Average Precision at K (AP@K)**: This is the precision calculated at the $K^{th}$ rank in the retrieved list, specifically considering only the top-K items. It is a useful measure in scenarios where the focus is on the relevance of the top part of the ranked list. The formula is as follows:

(20) $$\mathrm{AP}@K=\frac{\sum_{k=1}^{K}P\left(k\right)\times r\left(k\right)}{\min(K,\mathrm{Number}\;\mathrm{of}\;\mathrm{relevant}\;\mathrm{items})}$$

- *K* is the predefined number of top items to consider in the list.
- P(k) represents the precision at rank *k*.
-
$r\left(k\right)=\left\{\begin{array}{cc} 1 & \mathrm{if}\;\mathrm{the}\;\mathrm{item}\;\mathrm{is}\;\mathrm{relevant},\\ 0 & \mathrm{otherwise}. \end{array}\right.$

**mAP@K (Mean average precision at top-K ranks)**: This metric calculates the mean of the AP@K scores across all queries, providing a single measure that summarizes the effectiveness of a retrieval system at ranking relevant items within the top-K positions of the ranked list. It is defined as follows:

(21) $$\mathrm{mAP}@K=\frac{\sum_{q=1}^{\left|Q\right|}{\mathrm{AP}@K}_{q}}{\left|Q\right|}$$

where:

- |Q| is the cardinality of the query list.
- ${\mathrm{AP}@K}_{q}$ denotes the average precision at K for the $q^{th}$ query.

### 4.3. Performance Comparison

**Figure 3:**
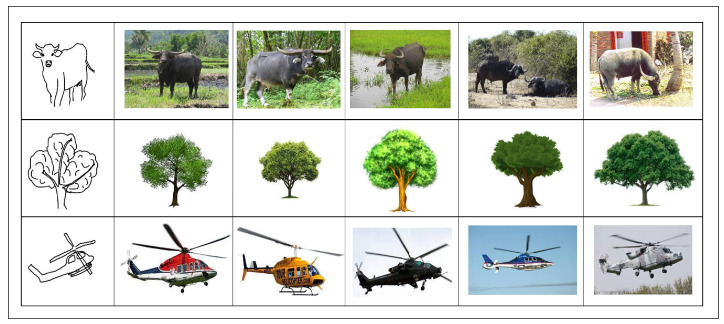
Retrieval results for the Sketchy Extended dataset. The image to the left is the input query sketch and the rest of the images are the retrieved images.

**Figure 4:**
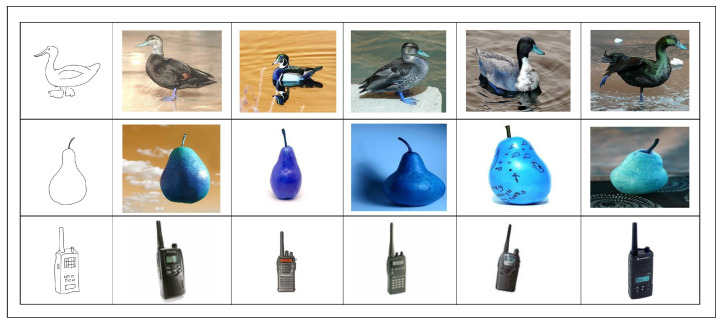
Retrieval results for the TU-Berlin extended dataset. The image to the left is the input query sketch and the rest of the images are the retrieved images.

The proposed methodology of ZS-SBIR, which employs the combination of StyleGen and SSiNN, is benchmarked against the current state-of-the-art approaches employed in the domain through extensive experimental evaluations. The experimental results, in comparison with existing approaches, for the TU-Berlin dataset, are presented in Table 3, while those for the Sketchy Extended dataset are detailed in Table 4. Our results clearly indicate that the ZS-SBIR method leveraging StyleGen and SSiNN exhibits superior performance metrics when compared to its contemporaries. The elevated effectiveness of our approach is evident, establishing a new performance benchmark in ZS-SBIR for the aforementioned datasets. Some of the retrieval results are presented in Figure 3 and Figure 4 for Sketchy Extended and TU-Berlin extended datasets, respectively.

**Robustness of the approach**: The datasets employed contain images and sketches that are notably diverse within each category. This diversity encompasses a wide range of drawing styles, levels of detail, and artistic interpretations, providing a robust foundation for evaluating the effectiveness of our method across varied real-world scenarios.
**Handling of zero-shot learning**: Based on the experimental findings, it is evident that our method outperforms current techniques in terms of performance. This enhanced performance can be attributed to two primary factors. Firstly, the implementation of the no-pair assumption within the StyleGen component significantly contributes to the model’s ability to generalize effectively, enabling the accurate generation of StyleGen images from previously unseen sketches. Secondly, the application of the stacked Siamese neural network (SSiNN) has been finely tuned to excel with zero-shot samples, further bolstering our method’s efficacy.
**Estimation of computation time:** The proposed methodology is implemented using the PyTorch framework [47] on a Windows PC with Intel Core I7 and Nvidia Geforce RTX. The models are built and optimized for 10 epochs, with Adam as the optimizer and a learning rate of $1\times 10^{-3}$ for the generator and $1\times 10^{-4}$ for the discriminator. The number of parameters in the generator network is 32,451, and that of the discriminator is 44,537. The time taken for running one epoch is an average of 8 h. So, the time taken for the one overall training cycle with 10 epochs is 80 h. In model training, it is often necessary to run through all training epochs multiple times to achieve optimal performance and robustness. This repetitive process allows the model to continually refine its parameters, learn from the dataset’s variability, and explore different solutions, improving generalization and stability. Considering this, the time taken for the overall model building will be in multiples of 80 h. Our approach to addressing the zero-shot sketch-based image retrieval (ZS-SBIR) problem by dividing it into two distinct stages—each focusing on a specific aspect of the challenge—is a strategic method that contributes to its superior performance. The following is a deeper analysis of why this method outperforms others, along with its limitations and potential areas for improvement:
**Strengths and reasons for superior performance:** Targeted problem-solving approach—in this approach, the ZS-SBIR problem is divided into two stages, with each focusing on a specific challenge—domain gap and knowledge gap. This allows for specialized strategies tailored to each aspect, potentially leading to more effective solutions.

- Stage 1—Domain gap solution with StyleGen framework: The first stage employs the StyleGen framework to specifically address the domain gap problem. By transforming sketches into a style more akin to the target images, we enhance feature compatibility, improving retrieval accuracy.
- Stage 2—Knowledge-gap solution with SSiNN: In the second stage, we utilize the stacked Siamese neural network (SSiNN) to tackle the knowledge gap problem.
- Separate optimization: By separately optimizing each stage, our approach achieves a higher degree of fine-tuning for each specific challenge, contributing to overall superior performance.

The overall performance of the proposed approach depends on the performance of the individual stages. Let $P_{\mathrm{overall}}$ be the overall system precision, $P_{\mathrm{stylegen}}$ be the precision of the StyleGen stage, and $P_{\mathrm{ssinn}}$ be the precision of the SSiNN stage. The performance of StyleGen in CBIR tasks [26] is as high as 99% for the CIFAR-10 dataset; however, for the datasets used in this work, it is around 94%, particularly attributed to the diverse nature of the classes in these datasets. A simplified estimate of the overall system efficacy can be represented as the product of the individual stages’ efficacies.

(22) $$P_{\mathrm{overall}}=P_{\mathrm{stylegen}}\times P_{\mathrm{ssinn}}$$

Despite the high performance of the retrieval block, the overall precision of the framework, as measured by mAP@all for the TU-Berlin extended dataset, is 59.4%, and for the Sketchy Extended dataset, it is 52.8%, as measured by mAP@200. The reason for the drop in the overall precision is attributed to the image equivalent representation conducted by the StyleGen block. The reason for this is the ambiguous nature of the sketches as discussed in the introduction section.

**Limitations and Areas for Improvement:**

- Complexity and resource intensity: The proposed two-stage process, while effective, is complex and resource-intensive compared to single-stage methods, which could be a limitation in terms of computational efficiency and practicality.
- Integration and cohesion between stages: Ensuring seamless integration and effective cohesion between the two stages is crucial. Any misalignment could potentially reduce the overall effectiveness.
- Cross-dataset generalization: Testing and refining our method on a broader range of datasets is a key focus, aiming to improve its generalizability and applicability to different real-world scenarios.
- Interpretability: While the approach demonstrates impressive performance in matching sketches to images, understanding the decision-making process of these models remains a challenge. This lack of transparency can be problematic, especially in applications where understanding the reasoning behind each match is crucial.

### 4.4. Ablation Studies

#### 4.4.1. Hyperparameter Selection

This subsection delves into the selection of the hyperparameters α and β from Equation (17), crucial for optimizing our model’s performance. It outlines the rationale and experimental process behind choosing specific values for these parameters. The choice of α=10 is based on the CycleGAN paper [34]. The parameter β is chosen based on experimental choice; experiments were conducted with three choices of β={0.5,5,10}. Based on the experimental results in Table 5, 0.5 was the better choice.

#### 4.4.2. Effectiveness of Identity Loss Function

The purpose of identity loss is to ensure that when an input from the target domain is provided to a generator, the output is identical or very close to the input, thereby preserving the original identity of the input in the absence of a domain shift. In this experiment, the effectiveness of the identity loss function in the overall performance is measured. The results demonstrate that the presence of identity loss marginally enhances the overall performance. For the TU-Berlin dataset, mAP@all increases from 58.8% to 59.4%, and for the Sketchy Extended dataset, mAP@200 increases from 52.3% to 52.8% when the identity loss function is considered in the overall loss function. The results are presented in Table 6.

#### 4.4.3. Retrieval Block Selection

To optimize this second stage, we conducted experiments with two distinct approaches for the retrieval block: one leveraging an autoencoder [48] and the other utilizing SSiNN [26]. The comparative analysis of these approaches, as detailed in our results in Table 7, clearly demonstrates that the SSiNN-based retrieval method significantly outperforms the autoencoder-based method.

## 5. Conclusions

In conclusion, our research successfully introduces a novel technique for ZS-SBIR that harnesses the potential of StyleGen and SSiNN (stacked Siamese neural networks). This approach has been empirically validated to outperform existing methods, marking a significant advancement in the field of content-based image retrieval. By ingeniously integrating the generative capabilities of StyleGen with the discriminative prowess of SSiNN, our method not only enhances the accuracy of zero-shot retrieval but also enriches the interpretability of the results. Our method effectively bridges the gap between sketches and photos, even in the absence of paired instances.

## Figures and Tables

**Table 1:** Network topology of the generator network.

Layer	Block	Configuration	Channels	Output Shape	Parameters
Convolution2D	Input Block	Filters: 64 Kernel Size: 7×7 Padding: (3,3), Reflect Stride: 1	In: 3 Out: 8	8×224×224	1184
InstanceNorm ReLU Activation	Input Block	none	In: 8 Out: 8	8×224×224	0
Convolution2D	Down Sampling Block 1	Filters: 128 Padding: (1,1) Kernel Size: 3×3 Stride: 2	In: 8 Out: 16	16×112×112	1168
InstanceNorm ReLU	Down Sampling Block 1	none	In: 16 Out: 16	16×112×112	0
Convolution2D	Down Sampling Block 2	Filters: 256 Kernel Size: 3×3 Padding: (1,1) Stride: 2	In: 16 Out: 32	32×56×56	4640
InstanceNorm ReLU	Down Sampling Block 2	none	In: 32 Out: 32	32×56×56	0
Convolution2D	Residual Block 1	Filters: 256 Kernel Size: 3×3 Padding: (1,1) Stride: 1	In: 32 Out: 32	32×56×56	9248
InstanceNorm ReLU	Residual Block 1	none	In: 32 Out: 32	32×56×56	0
Convolution2D	Residual Block 1	Filters: 256 Kernel Size: 3×3 Padding: (1,1), Reflect Stride: 1	In: 32 Out: 32	32×56×56	9248
InstanceNorm	Residual Block 1	none	In: 32 Out: 32	32×56×56	0
⋮	Residual Blocks 2 - 8	⋮	⋮	⋮	⋮
ConvTranspose 2D	Upsampling Block 1	Filters: 128 Kernel Size: 3×3 Padding: (1,1) Stride: 2	In: 32 Out: 16	16×112×112	4624
ConvTranspose 2D	Upsampling Block 2	Filters: 64 Kernel Size: 3×3 Padding: (1,1) Stride: 2	In: 16 Out: 8	8×224×224	1160
InstanceNorm ReLU	Upsampling Block 2	none	In: 8 Out: 8	8×224×224	0
Convolution 2D	Output Block	Filters: 3 Kernel Size: 7×7 Padding: (3,3), Reflect Stride: 1	In: 8 Out: 3	3×224×224	1179
Tanh	Output Block	none	In: 3 Out: 3	3×224×224	0

**Table 2:** Network topology of the discriminator network.

Layer	Kernel Size	Stride	Channels	Output Shape	Activation	Parameters
InstanceNorm ReLU	Upsampling Block 1	none	In: 16 Out: 16	16×112×112	0	
Conv 2D	4×4	2	In: 3 & Out: 64	112×112×64	LeakyReLU	392
Conv 2D	4×4	2	In: 64 & Out: 128	56×56×128	LeakyReLU	2064
Conv 2D	4×4	2	In: 128 & Out: 256	28×28×256	LeakyReLU	8224
Conv 2D	4×4	1	In: 256 & Out: 512	28×28×512	LeakyReLU	32,832
Conv 2D	4×4	1	In: 512 & Out: 1	28×28×1	LeakyReLU	1025

**Table 3:** Performance comparison of StyleGen plus the SSiNN methodology compared to existing approaches for the TU-Berlin dataset.

Approach	mAP@all	AP@100
Zero-shot sketch image hashing [37]	22.0	29.1
Content style decomposition [38]	25.4	35.5
Semantically tied paired cycle consistency [39]	29.3	39.2
OCEAN [40]	33.3	46.7
Domain smoothing network [41]	48.1	58.6
Progressive domain-independent feature decomposition network [42]	48.3	60.0
Norm-guided adaptive visual embedding [43]	49.3	60.7
Relationship-preserving knowledge distillation [44]	48.6	61.2
ACNet [23]	57.5	65.8
Proposed approach	59.4	66.3

**Table 4:** Performance comparison between the StyleGen plus SSiNN methodology and the existing approaches for the Sketchy Extended dataset.

Approach	mAP@200
Conditional variational autoencoder [22]	22.5
Content style decomposition [38]	35.8
Doodle [45]	47.0
Semantic-aware knowledge preservation [46]	49.7
Relationship-preserving knowledge distillation [44]	50.2
ACNet [23]	51.7
Proposed approach	52.8

**Table 5:** Experimental results for the selection of hyperparameters for the datasets.

α	β	Sketchy Extended mAP@200	TU-Berlin Extended mAP@all
10.0	10.0	47.7	55.6
10.0	5.0	49.5	59.1
10.0	0.5	52.8	59.4

**Table 6:** Experimental results for the assessment of the effectiveness of identity loss function.

Dataset	Metric	Without $L_{\mathrm{identity}}$	With $L_{\mathrm{identity}}$
TU-Berlin Extended	mAP@all	58.8	59.4
Sketchy Extended	mAP@200	52.3	52.8

**Table 7:** Experimental results for the selection of the retrieval block.

Dataset	Metric	Autoencoder	SSiNN
TU-Berlin Extended	mAP@all	46.1	59.4
Sketchy Extended	mAP@200	39.9	52.8

## Data Availability

The original data presented in the study are openly available at https://dl.acm.org/doi/10.1145/2897824.2925954 (accessed on 1 October 2023), https://cybertron.cg.TU-Berlin.de/eitz/projects/classifysketch/ (accessed on 1 October 2023) and https://www.image-net.org/ (accessed on 1 October 2023).
